# Factors associated with growth disturbance at celiac disease diagnosis in children: A retrospective cohort study

**DOI:** 10.1186/s12876-015-0357-4

**Published:** 2015-10-06

**Authors:** Samuli Nurminen, Laura Kivelä, Juha Taavela, Heini Huhtala, Markku Mäki, Katri Kaukinen, Kalle Kurppa

**Affiliations:** 1 School of Medicine, University of Tampere, FIN-33014 Tampere, Finland; 2 Tampere Center for Child Health Research, University of Tampere and Tampere University Hospital, Tampere, Finland; 3 School of Health Sciences, University of Tampere, Tampere, Finland; 4 Department of Internal Medicine, Tampere University Hospital, Tampere, Finland

**Keywords:** Celiac disease, Children, Growth failure

## Abstract

**Background:**

Impaired growth is a well-known complication in celiac disease, but factors associated with it are poorly known. We investigated this issue in a large cohort of children.

**Methods:**

530 children with biopsy-proven celiac disease were included. The participants were divided into two groups on the basis of the presence (n = 182) or absence (n = 348) of growth disturbance at diagnosis. Histological, serological and clinical characteristics were compared between children with growth failure and those with normal growth. Further, patients with growth failure as the sole clinical presentation were compared to those with poor growth and concomitant other symptoms.

**Results:**

Children with growth failure were younger (*p* < 0.001) and had lower hemoglobin (*p* = 0.016) and higher celiac antibody (*p* < 0.001), alanine aminotransferase (*p* = 0.035) and thyroid-stimulating hormone values (*p* = 0.013) than those with normal growth. Significantly associated with growth failure at diagnosis were age <3 years (OR 4.3 (95 % CI 2.5-7.5) *vs* older age), diagnosis before the year 2000 and in 2000–09 (OR 3.1 (1.8-5.4) and OR 1.8 (1.1-2.8) *vs* diagnosis in 2010–2013), presence of total and subtotal villous atrophy (OR 4.2 (2.5-7.0) and OR 2.0 (1.3-3.2) *vs* partial atrophy), severe symptoms (OR 3.4 (1.8-6.7) vs mild symptoms) and vomiting (OR 3.1 (1.5-6.3). The presence of abdominal pain reduced the risk (OR 0.5 (0.3-0.7)), while there was no effect of gender, diarrhea, constipation, other chronic diseases and celiac disease in the family. Children evincing poor growth as the sole clinical presentation were older (*p* < 0.001) and had higher hemoglobin (*P* < 0.001) and total iron (*p* = 0.010) values and lower TG2ab values (*p* = 0.009) than those with growth disturbance and other symptoms.

**Conclusions:**

In particular young age and severe clinical and histological presentation were associated with growth disturbance at celiac disease diagnosis. Children with only poor growth are markedly different from those with other concomitant symptoms, suggesting different pathogenic mechanisms.

## Background

Population-based screening studies have revealed celiac disease to be a very common health problem with a true prevalence of up to 3 % in children [1]. Besides the classical gastrointestinal symptoms, diarrhea and malabsorption, the condition may present with various extraintestinal manifestations [2]. A frequent, and sometimes the only clinical presentation is impaired growth [3, 4]. Despite being a long-recognized sign of the disease the pathophysiology and factors accompanying growth failure in celiac disease remain somewhat obscure. Traditionally the poor growth has been attributed to severe histological damage leading to malabsorption of essential nutrients, but the actual evidence for this is scant [5]. Other proposed mechanisms are for example abnormalities in the growth hormone/insulin-like growth factor-1 axis [6–8] and increased prevalence of anti-pituitary antibodies [9]. Regardless of the cause, after the commencement of a gluten-free diet significant catch-up growth usually follows [10]. However, in particular in those diagnosed in later childhood this may remain incomplete, leading possibly to reduced adult height [10], though this aspect remains controversial [11, 12]. The heterogeneous clinical picture of celiac disease makes it difficult to recognize and predisposes to long diagnostic delay [13], further increasing the risk of permanent growth failure. In order to prevent this complication it would be important to better understand the factors associated with poor growth in celiac disease.

In Finland celiac disease is common, and there have been nationwide guidelines for its diagnosis and treatment since the end of the 1990s [14]. A major aim of the regularly updated guidelines is early diagnosis and subsequent prevention of the possible severe complications, including poor growth in childhood. We here investigated the prevalence and associated factors in growth disturbance in pediatric celiac disease in the era of the modern diagnostic approach.

## Methods

### Patients and data collection

The study was conducted at the Tampere Center for Child Health Research, University of Tampere and Tampere University Hospital. The study cohort comprised 530 children (age under 16 years at diagnosis) with biopsy-proven celiac disease collected in our regularly updated pediatric research database. The patient information was either updated prospectively or collected from the medical records and, if incomplete, later supplemented with personal or parents’ interviews by a study nurse with expertise in celiac disease. The following information was gathered from all study subjects: demographic and anthropometric data, year of the celiac disease diagnosis, clinical presentation at diagnosis, values of serum celiac disease-specific antibodies and various laboratory parameters, severity of small-bowel mucosal damage and presence of either known celiac disease-associated or other concomitant chronic disease (e.g. type 1 diabetes mellitus, autoimmune thyroidal disease, asthma, allergies) and celiac disease in the family. After the analyses the children were divided into subjects with poor growth and those with normal growth prior to the celiac disease diagnosis (see below in detail).

### Clinical symptoms

The severity of the clinical presentation at celiac disease diagnosis was further classified into four categories as follows: no symptoms (asymptomatic screen-detected subjects), mild symptoms (occasionally disturbing gastrointestinal or extraintestinal symptoms), moderate symptoms (multiple or more disturbing or frequent symptoms) and severe symptoms (symptoms seriously disturbing normal life; e.g. continuous symptoms or night awakenings due to the symptoms). Gastrointestinal symptoms were sub-categorized into diarrhea, abdominal pain, constipation and vomiting.

### Serology and laboratory parameters

In our hospital the serum transglutaminase 2 (TG2ab) antibodies are measured using automatized human recombinant-based EliA assay (Phadia AB, Uppsala, Sweden). A TG2ab value of 7 U/l or higher is considered positive and the maximum reported value is 120 U/l. Serum endomysial antibodies (EmA) are measured in our research center by an indirect immunofluorescence-based in-house method using human umbilical cord as substrate [15]. An EmA dilution of 1:≥5 is considered positive and positive sera are further diluted from 1:50 to 1:4000.

The following laboratory values were collected from each child at diagnosis when available: blood hemoglobin (g/l), serum total iron (μmol/l), the mean corpuscular volume (MCV) (fl), serum alkaline phosphatase (U/l), serum alanine aminotransferase (ALT) (U/l), serum albumin (g/l), serum thyroid-stimulating hormone (TSH) (mU/l) and serum thyroxin (pmol/l). Anemia at diagnosis was defined as a blood hemoglobin value lower than that of the age- and gender-specific reference in the local hospital laboratory_._

### Small-bowel mucosal morphology

In our clinical practice a minimum of four duodenal samples are taken upon upper gastrointestinal endoscopy in all cases of celiac disease suspicion. The biopsies are passed to the pathology unit, where they are processed and analyzed. Only correctly oriented specimens are accepted for further microscopic analyses [16]. The severity of small-bowel mucosal villous atrophy here was categorized based on the hospital pathologist’s original grading into partial (PVA), subtotal (SVA) and total villous atrophy (TVA). These correspond approximately to Marsh-Oberhuber grades IIIa, IIIb and IIIc.

### Growth parameters

Height-for-age was expressed in standard deviation (SD) units, which compare the height of a child to the average height of Finnish children of the same age [17]. Poor growth was defined as an abnormal deceleration of growth development compared with age- and gender-specific reference values or growth below the of expected target height based on the mean of parental heights [18]. The expected growth rate was considered abnormally low if the current height differed from the expected more than −2.3 SD. If the parental heights were not known, the child’s height was allowed to differ a maximum of −2.7 SD from the age- and sex-based reference [17]. In most cases the presence of poor growth was defined by the physician referring the patient for further endoscopic investigations. The weight of the children was expressed as weight-for-height percentage, as age- and gender-matched SD units and as body mass index (BMI, kg/m^2^).

### Ethical aspects

Collection of the medical records and patient interviews were approved by the Department of Pediatrics, Tampere University Hospital and by the Ethics Committee of the Pirkanmaa Hospital District, Tampere, Finland. Written informed consent was obtained from all patients and/or their parents participating in the personal interviews.

### Statistical analysis

Statistical analyses were performed using Statistical Package for the Social Sciences (SPSS) statistical software (SPSS Inc., Chicago, IL, USA). In Tables 1 and 4 the data are expressed either in medians with upper and lower quartiles (Q1, Q3) or as percentages and differences between the growth failure and normal growth groups were compared with Mann–Whitney *U* test or Chi square test. In Tables 2 and 3 the relative risk of growth failure is expressed using odds ratios with 95 % confidence intervals. A *P* value < 0.05 was considered statistically significant.

**Table 1:** Baseline characteristics in 530 children with or without growth failure before celiac disease diagnosis

		Growth failure *n* = 182		Normal growth *n* = 348	
	*n* ^a^	Median (Q_1,_ Q_3_)	*n* ^a^	Median (Q_1,_ Q_3_)	*P* value
Age at diagnosis, yr	182	6.3 (2.5, 11.7)	348	8.0 (5.0, 12.0)	<0.001
Age of menarche, yr	23	13.0 (12.0, 13.0)	45	12.0 (12.0, 13.0)	0.146
Height, SD^b^	45	−0.5 (−1.2, 0.4)	134	0.2 (−0.5, 0.9)	0.004
Weight, SD	34	−1.1 (−1.9, −0.2)	114	−0.3 (−0.9, 0.6)	0.003
Weight/height percentage	65	−9.0 (−15.0, −1.0)	163	0.1 (−8.0, 9.0)	<0.001
Body mass index, kg/m^2^	68	15.7 (14.3, 16.6)	155	16.6 (15.1, 19.2)	<0.001
EmA, titer	81	500 (200, 2000)	205	200 (100, 1000)	<0.001
TG2ab, U/l^c^	98	120.0 (71.3, 120.0)	253	120.0 (39.0, 120.0)	0.035
Hemoglobin, g/l	129	122 (115, 128)	248	126 (115, 133)	0.016
MCV, fl	96	81 (75, 83)	207	81 (76, 83)	0.662
Alkaline phosphatase, U/l	42	309 (148, 511)	79	231 (184, 322)	0.180
ALT, U/l	34	24 (18, 32)	83	20 (16, 25)	0.035
Total iron, μmol/l	33	10.0 (6.6, 15.0)	58	12.5 (9.3, 17.8)	0.304
Albumin, g/l	18	37 (34, 41)	50	39 (37, 41)	0.252
TSH, mU/l	43	2.8 (2.2, 3.7)	58	2.4 (1.6, 3.1)	0.013
Thyroxin, pmol/l	31	14.4 (12.6, 16.9)	25	14.8 (12.9, 16.3)	0.817

Q_1_, Q_3_, lower and upper quartiles; SD, standard deviation; EmA, endomysial antibodies; TG2ab, transglutaminase 2 antibodies; MCV, mean corpuscular volume; ALT, alanine aminotransferase; TSH, thyroid stimulating hormone

^a^Data available

^b^Includes only measurements made by a pediatrician at the time of gastrointestinal endoscopy

^c^Upper limit of the assay 120.0 U/l

**Table 2:** Association between clinical characteristics and growth disturbance at diagnosis in 530 children with celiac disease

	*n* ^a^	Growth failure, %	Odds ratio	95 % CI	*P* value
Gender					
Boys	194	33.5	1		
Girls	336	34.8	1.06	0.73 - 1.54	0.759
Age at diagnosis, years					
<3	74	66.2	4.32	2.49 - 7.48	<0.001
3-7	203	26.6	0.80	0.53 - 1.20	0.281
8-17	253	31.2	1		
Year of diagnosis					
<2000	114	48.2	3.11	1.82 - 5.35	<0.001
2000-09	277	34.3	1.75	1.10 - 2.78	0.019
2010-13	139	23.0	1		
Type 1 diabetes mellitus					
No	475	33.7	1		
Yes	41	24.4	0.64	0.30 - 1.33	0.228
Thyroid disease					
No	502	32	1		
Yes	13	54	2.45	0.81 - 7.40	0.113
No	474	32.9	1		
Yes	42	33.3	1.02	0.52 - 1.99	0.956
History of allergy					
No	393	34.9	1		
Yes	125	28.0	0.73	0.47 - 1.13	0.157
Other concomitant chronic disease^b^					
No	202	29.7	1		
Yes	315	35.6	1.31	0.89 - 1.91	0.169
First-degree relative with celiac disease					
No	157	35.7	1		
Yes	140	26.4	0.65	0.39 - 1.07	0.087

^a^Data available

^b^E.g. epilepsy, rheumatoid arthritis, psychiatric disorder, inflammatory bowel disease, congenital heart disease, immune deficiency

**Table 3:** Association between histologic and symptomatic presentation and growth disturbance

	*n* ^a^	Growth failure, %	Odds ratio	95 % CI	*P* value
Degree of small-bowel mucosal villous atrophy					
Partial	174	20.7	1		
Subtotal	194	34.5	2.02	1.26 - 3.24	0.003
Total	126	52.4	4.22	2.54 - 7.00	<0.001
Severity of symptoms^b^					
Mild	258	28.7	1		
Moderate	107	35.5	1.37	0.85 - 2.21	0.198
Severe	43	58.1	3.45	1.78 - 6.70	<0.001
Asymptomatic	121	37.2	1.47	0.93 - 2.33	0.097
Diarrhea					
No	319	32.3	1		
Yes	181	37.6	1.26	0.86 - 1.85	0.232
Abdominal pain					
No	267	42.7	1		
Yes	230	25.7	0.46	0.32 - 0.68	<0.001
Constipation					
No	425	35.8	1		
Yes	72	26.4	0.64	0.37 - 1.13	0.124
Vomiting					
No	466	32.4	1		
Yes	32	59.4	3.05	1.47 - 6.34	0.003
Anemia					
No	420	32.4	1		
Yes	91	37.4	1.25	0.78 - 2.00	0.361

^a^Data available

^b^Growth failure was not considered as a symptom here at diagnosis in 530 children with celiac disease

**Table 4:** Comparison of baseline data between celiac children with poor growth as sole clinical presentation and those with other concomitant symptoms

	*n* ^a^	Poor growth as only symptom *n* = 46	*n* ^a^	Poor growth with other symptoms *n* = 136	*P* value
Age at dg, median, yr	46	11.5 (7.4, 13.1)	136	5.0 (2.0, 10.0)	<0.001
Girls, %	46	54	136	67	0.104
Anemia, %	40	15	130	22	0.085
Degree of villous atrophy, %					0.449
Partial	10	23	26	21	
Subtotal	14	32	53	42	
Total	20	45	46	37	
Body mass index, kg/m^2^	24	15.5 (14.3, 17.0)	44	15.8 (14.3, 16.5)	0.847
Hemoglobin, g/l	28	128 (123, 135)	101	120 (110, 127)	<0.001
MCV, fl	23	82 (78, 85)	73	79 (74, 83)	0.039
Alkaline phosphatase	10	284 (119, 640)	32	320 (148, 511)	0.859
Total iron, μmol/l	8	19.5 (9.9, 34.2)	25	7.5 (5.6, 14.2)	0.010
TSH, mU/l	12	3.1 (1.8, 3.8)	31	2.7 (2.0, 3.6)	0.684
Thyroxin, pmol/l	8	15.9 (14.3, 18.1)	23	14.2 (12.5, 16.4)	0.298
EmA, titer	26	500 (200, 1250)	81	1000 (200, 4000)	0.516
TG2ab, U/l	31	120 (30, 120)	67	120 (95, 120)	0.009

^a^Data available

SD, standard deviation; MCV, mean corpuscular volume; TSH, thyroid stimulating hormone; EmA, endomysial antibodies; TG2ab, transglutaminase 2 antibodies

## Results

Altogether 182 (34 %) children presented with disturbed growth and 348 (66 %) with normal growth at celiac diagnosis. Children with poor growth were significantly younger and had by definition lower median height but also lower weight parameters compared with those with normal growth (Table 1). There was also a trend towards later onset of menarche in girls with growth failure, but this was not significant (Table 1).

Serum EmA and TG2ab values were significantly higher in the growth failure group than in the normal growth group at diagnosis (Table 1). Further, median hemoglobin was significantly lower and ALT and TSH higher in children with poor growth. TSH was above the reference in 4 children with growth disturbance and in 2 with normal growth; however, none of these 6 had TSH values >10 mU/l or other clinical signs of hypothyroidism such as fatigue, increasing weight or weakness. No differences in thyroxin values or other laboratory parameters were observed between the groups (Table 1). Previously diagnosed concomitant thyroid disease under treatment was noted in 7 (4.1 %) children with poor growth and 6 (1.7 %) with normal growth (*p* = 0.102).

Among the demographic and clinical characteristics significantly associated with growth disturbance at celiac disease diagnosis was age below three years compared with older age and celiac disease diagnosis before the year 2010 compared with the later era (Table 2). There was no association between growth failure and gender, presence of any other concomitant chronic disease or presence of celiac disease in the family (Table 2).

A significant association was seen between abnormal growth and the presence of subtotal and total small-bowel mucosal villous atrophy (Table 3). Of symptoms overall the presence of in general severe symptoms and of specific gastrointestinal symptoms vomiting increased the risk of poor growth. In contrast, abdominal pain reduced the risk, whereas the presence of diarrhea, constipation and anemia had no effect (Table 3).

In a separate analysis among the 182 children with growth failure this was the sole clinical presentation of celiac disease in 46 (25 %) subjects at diagnosis, while the remaining 136 (75 %) also had other clinical symptoms (Table 4). Children with poor growth as the sole manifestation were significantly older and had higher hemoglobin, MCV and total iron values and lower TG2ab values than those with other concomitant symptoms, while there were no significant differences between the groups in the other study variables (Table 4).

## Discussion

The present study demonstrated that children with poor growth at celiac disease diagnosis are significantly younger and have more severe disease in terms of symptoms, serology and histological damage compared with those with normal growth. It was also shown that the risk of growth failure has decreased during the past few decades, and that children with growth failure as the sole presentation are markedly different from those with concomitant other symptoms.

The association between histology and growth was demonstrated by the more frequent observation of poor growth in children with subtotal or total villous atrophy compared with those with partial atrophy. Although a seemingly logical finding, there is a surprising scarcity of studies touching upon this issue, and the few conducted have shown no clear relationship [19, 21, 22]. This discrepancy between the present and earlier results might be explained by the smaller numbers of patients in previous studies and variable definitions of growth failure. Weizman and colleagues [19] also speculated that the extent of mucosal damage might be more related to poor growth in celiac disease than the degree of atrophy; this is however, contradicted by evidence that the extent of enteropathy does correlate with clinical presentation [22]. Our findings support the role of villous atrophy and malabsorption of essential nutrients as an important cause of decreased growth in celiac disease. In accord with this was the observation of lower weight and hemoglobin values in the growth failure group. Similarly, even though still mostly at normal level, the ALT values were significantly higher level in children with poor growth, again indicating more advanced disease.

Here children with poor growth had significantly higher celiac antibody levels at diagnosis than those with normal growth. Previously Bingley and colleagues [23] have shown EmA-positive children to be shorter and lighter than corresponding seronegative non-celiac children. To our knowledge, however, the difference in serology noted here in children with celiac disease has not been reported before. The finding is in accord with the previously shown association between higher antibody levels and more severe histological and clinical presentation [20, 24, 25]. The correlation between serology and poor growth might thus in fact be a result of more severe mucosal damage, but it is also possible that the antibodies have a direct role in the disease pathogenesis [26].

The presence of growth failure was also associated with generally more severe gastrointestinal symptoms. Of specific symptoms vomiting increased the risk, while diarrhea, somewhat unexpectedly, did not. In contrast, the presence of the “non-classical” symptom abdominal pain in fact reduced the risk. Severity of gastrointestinal symptoms has previously been linked with more advanced villous atrophy in some studies [19, 24] but not in others [27, 28]. Interestingly, adult celiac disease patients with diarrhea were recently shown to have less severe villous atrophy than those with anemia [29]. Although in that study the possible confounding diseases such as microscopic colitis were not systemically excluded, it nicely demonstrates the complicated relationship between clinical and histological presentation in celiac disease. It is also possible to have growth-disturbing protein-losing enteropathy without diarrhea [30]. The protective effect of abdominal pain might be explained for example by less severe villous atrophy in these subjects. Obviously more studies are needed to decipher the mechanisms behind the multifaceted clinical picture in celiac disease.

Children with poor growth were almost two years younger than those with normal growth, and at particular risk were those below three years at diagnosis. Similar results have previously been reported from Sweden [31]. These findings suggest that the rapid growth during infancy is particularly vulnerable to malabsorption caused by untreated celiac disease. Conversely, it is possible that toddlers with poor growth as a presenting symptom are diagnosed earlier since they are regularly followed in child welfare clinics. Interestingly, children with growth failure as the sole presentation were significantly older than those with concomitant other symptoms. There could be different pathophysiological mechanisms present, and it has indeed been speculated that poor growth in children with classical symptoms is caused by malabsorption and in those with atypical presentation by abnormalities in the growth hormone-insulin-like growth factor axis [6–8, 32]. Also, as presumed in Crohn’s disease [33, 34], mucosal inflammation may directly contribute to abnormal growth. Because of the increased risk of growth hormone deficiency, there should be low threshold for endocrinological evaluation in children with no significant catch-up growth after one year on a gluten-free diet.

Interestingly, the TSH values here were higher in the growth failure group. Children with celiac disease are known to be at increased risk of autoimmune thyroid diseases [34]. This notwithstanding, only four children with growth failure had TSH values above the reference and none of them had other hypothyreosis symptoms or values usually required for such a diagnosis [35]. It is still possible that subclinical hypothyreosis had an exacerbating effect on the poor growth, but currently it remains controversial whether these children would benefit from hormone replacement therapy [36]. In any case, it is important to remember the possibility of associated thyroid disease in poorly growing children with celiac disease.

The finding that risk of growth failure was decreased over time is in line with other recent reports [4, 31, 37] and very likely reflects earlier diagnosis due to increased awareness and improved diagnostic tools. We found a higher prevalence of poor growth than previously reported by Savilahti and colleagues [4] but lower than that reported by group under Rashid [38]. These discrepancies might be explained by differences in definition, as growth failure has often been defined as a height below −2 SD compared with the reference population (“short stature”) [4], whereas we defined it as either height below the expected target or abnormal deflection of the growth. The latter method has proved effective and may allow indentification children with celiac disease earlier and thus reduce the risk of permanent growth failure [19, 39]. In contrast, short stature as such is often only a normal variant and thus not a particularly reliable marker for true growth abnormality [40].

The strengths of this study were its large size and well-defined celiac disease diagnoses. We also had a wide range of clinically relevant data on each patient. Nevertheless, in particular laboratory values were lacking in a substantial part of the study subjects. Another limitation was that, although proved appropriate, the definition of growth failure used here might limit generalization of the results. Finally, since growth hormone metabolism or other endocrinological factors possibly implicated were not investigated here, we cannot verify the mechanisms underlying poor growth, this being an issue for future studies.

## Conclusions

To conclude, our results demonstrate that growth failure in symptomatic children with celiac disease is associated in particular with young age and severe presentation at diagnosis. Differences between children having poor growth as the sole clinical presentation and those with concomitant other symptoms indicate that there are different underlying mechanisms.
